# (4S*)-2-Methyl­amino-3-nitro-4-(4-nitro­phen­yl)-5,6,7,8-tetra­hydro-4*H*-chromen-5-one

**DOI:** 10.1107/S1600536813021181

**Published:** 2013-08-03

**Authors:** P. Narayanan, Jayabal Kamalraja, Paramasivam T. Perumal, K. Sethusankar

**Affiliations:** a Department of Physics, RKM Vivekananda College (Autonomous), Chennai 600 004, India; b Organic Chemistry Division, Central Leather Research Institute, Adyar, Chennai 600 020, India

## Abstract

The title compound, C_16_H_15_N_3_O_6_, is asymmetric with a chiral centre located in the pyran ring and crystallizes as a racemate. The six-membered carbocyclic ring adopts an envelope conformation with the central CH_2_ C atom as the flap. The amine N atom deviates from the mean plane of the pyran ring by 0.1365 (15) Å. The nitro­phenyl ring is almost orthogonal to the pyran ring and the mean plane of the six-membered carbocyclic ring, the dihedral angle between their mean planes being 88.30 (7) and 87.61 (8)°, respectively. The mol­ecular structure is stabilized by an intra­molecular N—H⋯O hydrogen bond, which generates an *S*(6) ring motif. In the crystal, mol­ecules are linked *via* C—H⋯O hydrogen bonds, forming infinite bands lying parallel to (-110) and composed of alternate *R*_2_^2^(24) and *R*_2_^4^(12) graph-set ring motifs. The crystal structure is further stabilized by C—H⋯π inter­actions, forming a three-dimensional structure.

## Related literature

For the uses and biological importance of chromene, see: Ercole *et al.* (2009 ▶); Geen *et al.* (1996 ▶); Khan *et al.* (2010 ▶); Raj *et al.* (2010 ▶). For related structures, see: Narayanan *et al.* (2013*a* ▶,*b* ▶). For graph-set notation, see: Bernstein *et al.* (1995 ▶). For puckering parameters, see: Cremer & Pople (1975 ▶).
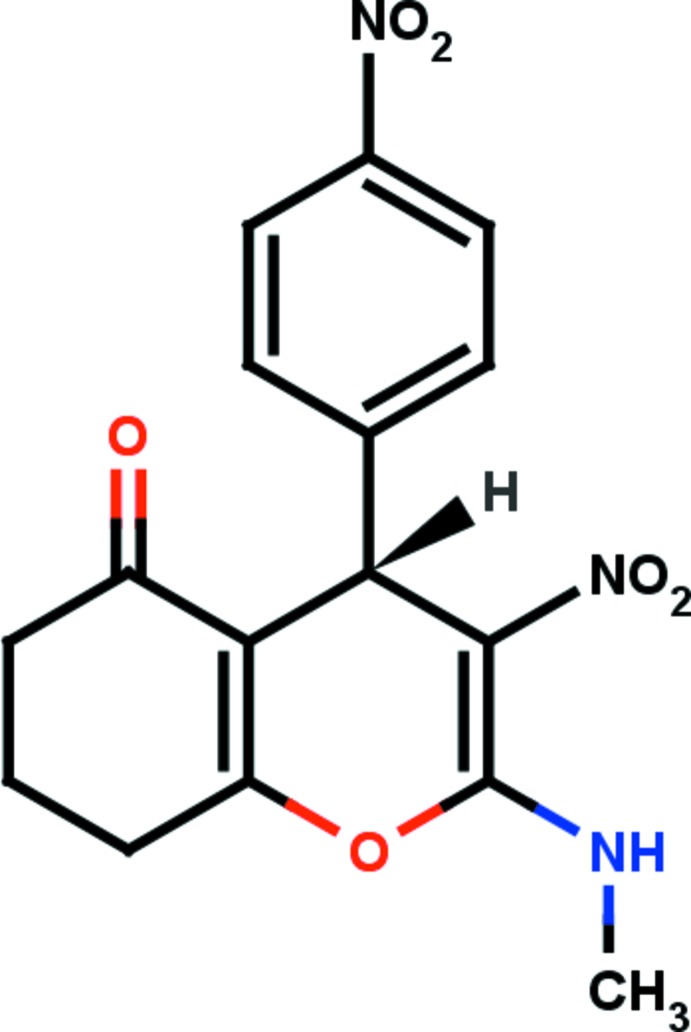


## Experimental

### 

#### Crystal data

C_16_H_15_N_3_O_6_
*M* *_r_* = 345.31
Triclinic, 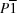

*a* = 8.2587 (3) Å
*b* = 8.7679 (4) Å
*c* = 10.9346 (5) Å
α = 101.616 (2)°
β = 90.426 (2)°
γ = 91.930 (2)°
*V* = 775.05 (6) Å^3^
*Z* = 2
Mo *K*α radiation
μ = 0.12 mm^−1^
*T* = 296 K
0.30 × 0.25 × 0.20 mm

#### Data collection

Bruker SMART APEXII CCD diffractometer
Absorption correction: multi-scan (*SADABS*; Bruker, 2008 ▶) *T*_min_ = 0.966, *T*_max_ = 0.977
10840 measured reflections
3161 independent reflections
2633 reflections with *I* > 2σ(*I*)
*R*_int_ = 0.026

#### Refinement

*R*[*F* ^2^ > 2σ(*F* ^2^)] = 0.044
*wR*(*F* ^2^) = 0.126
*S* = 1.04
3161 reflections
230 parameters
1 restraint
H atoms treated by a mixture of independent and constrained refinement
Δρ_max_ = 0.24 e Å^−3^
Δρ_min_ = −0.31 e Å^−3^

### 

Data collection: *APEX2* (Bruker, 2008 ▶); cell refinement: *SAINT* (Bruker, 2008 ▶); data reduction: *SAINT*; program(s) used to solve structure: *SHELXS97* (Sheldrick, 2008 ▶); program(s) used to refine structure: *SHELXL97* (Sheldrick, 2008 ▶); molecular graphics: *ORTEP-3 for Windows* (Farrugia, 2012 ▶) and *Mercury* (Macrae *et al.*, 2008 ▶); software used to prepare material for publication: *SHELXL97* and *PLATON* (Spek, 2009 ▶).

## Supplementary Material

Crystal structure: contains datablock(s) global, I. DOI: 10.1107/S1600536813021181/su2627sup1.cif

Structure factors: contains datablock(s) I. DOI: 10.1107/S1600536813021181/su2627Isup2.hkl

Supplementary material file. DOI: 10.1107/S1600536813021181/su2627Isup3.cml

Additional supplementary materials:  crystallographic information; 3D view; checkCIF report

## Figures and Tables

**Table 1:** Hydrogen-bond geometry (Å, °) *Cg*1 is the centroid of the C1–C6 ring.

*D*—H⋯*A*	*D*—H	H⋯*A*	*D*⋯*A*	*D*—H⋯*A*
N1—H1*A*⋯O3	0.90 (3)	1.89 (3)	2.601 (2)	134 (2)
C11—H11*A*⋯O6^i^	0.97	2.54	3.352 (2)	141
C11—H11*B*⋯O5^ii^	0.97	2.54	3.186 (2)	124
C10—H10*A*⋯*Cg*1^iii^	0.97	2.90	3.515 (2)	135
C16—H16*B*⋯*Cg*1^iv^	0.96	2.90	3.577 (3)	142

Symmetry codes: (i) 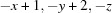; (ii) 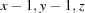; (iii) 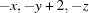; (iv) 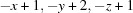.

**Fig. 1.**
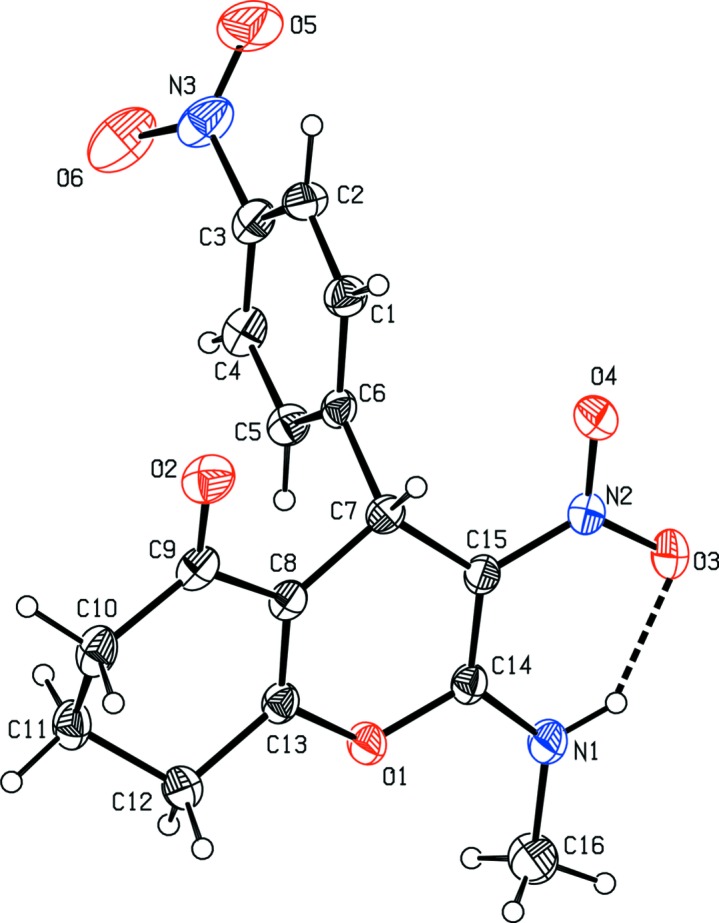
The molecular structure of the title molecule, with atom labelling. The displacement ellipsoids are drawn at the 30% probability level. The intramolecular N-H···O hydrogen bond, which generates an S(6) ring motif, is shown as a dashed line (see Table 1 for details).

**Fig. 2.**
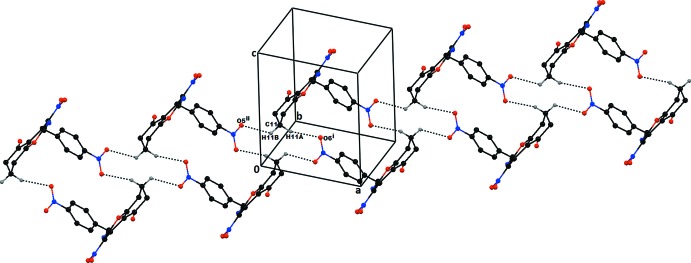
The crystal packing of the title compound, with the C-H···O hydrogen bonds shown as dashed lines (see Table 1 for details; symmetry codes: (i) -x+1, -y+2, -z; (ii) x-1, y-1, z).

## supplementary crystallographic information

### 1. Comment

Chromene derivatives are very important heterocyclic compounds that have a variety of industrial, biological and chemical synthesis applications (Geen *et al.*, 1996; Ercole *et al.*, 2009). They exhibit a number of pharmacological activities such as anti-HIV, anti-inflammatory, anti-bacterial, anti-allergic, anti-cancer (Khan *et al.*, 2010; Raj *et al.*, 2010). Against this background, the X-ray analysis of the title compound has been carried out to study its structural aspects.

The title compound, Fig. 1, consists of a chromene moiety attached to a nitrophenyl ring, a nitro group and a methylamine group. The molecular structure is stabilized by an intra molecular N1—H1A···O3 interaction, which generates an *S*(6) ring motif (Table 1 and Fig. 1). The pyran ring (C7/C8/C13-C15/O1) mean plane is almost orthogonal to the nitrophenyl ring (C1–C6), with a dihedral angle of 88.30 (7) °.

The pyran ring is almost coplanar with the mean plane of the nitro group (N2/O3/O4), with a dihedral angle of 3.99 (11)°. The mean plane of the six membered carbocyclic ring (C8–C13) makes a dihedral angle of 87.61 (8) ° with the nitrophenyl ring (C1–C6), again they are almost perpendicular to each other.

The six membered carbocyclic rings (C8–C13) of the chromene moiety adopts an *envelope* conformation on atom C11 with puckering parameters (Cremer & Pople, 1975): Q_2_ = 0.4018 (17) Å, Q_3_ = -0.2465 (18) Å and φ_2_ = 358.1 (3). Atom C11 deviates from the mean plane of the rest of the ring atoms by 0.3325 (18) Å. The amine group nitrogen atom N1 deviates by -0.1365 (15) Å from the mean plane of the pyran ring. The title compound exhibits structural similarities with already reported related structures (Narayanan *et al.*, 2013*a*,*b*).

In the crystal, molecules are linked *via* C-H···O hydrogen bonds (Table 1 and Fig. 2), which form infinite bands lying parallel to plane (-1 1 0), and which enclose alternate *R*_2_^2^(24) and *R*_2_^4^(12) graph-set ring motifs (Bernstein *et al.*, 1995). The crystal structure is further stabilized by C-H···\p interactions (Table 1) forming a three-dimensional structure.

### 2. Experimental

A solution of the requisite aldehyde (0.151 g, 1.0 mmol), cyclic 1,3-dicarbonyl compound (0.112 g, 1.0 mmol), NMSM (0.148 g, 1.0 mmol) and piperidine (0.2 equiv) in EtOH (2 ml) was stirred for 1.5 hrs. After the reaction was complete as indicated by TLC, the product was filtered and washed with EtOH (2 ml) to remove the excess base and other impurities. Single crystals suitable for X-ray diffraction were prepared by slow evaporation of a solution of the title compound in ethanol at room temperature.

### 3. Refinement

Positions of the hydrogen atoms were localized from difference electron density maps. The H-atoms of the amine group were refined with distance restraints of N—H = 0.90 (1) Å with *U*_iso_(H) = 1.2*U*_eq_(N). The C-bound H atoms were treated as riding atoms: C-H = 0.93, 0.97, 0.98 and 0.96 Å for CH(aromatic), CH_2_, CH and CH_3_ H atoms, respectively, with *U*_iso_(H) = 1.5*U*_eq_(C-methyl) and = 1.2*U*_eq_(C) for other H atoms. The rotation angles for methyl groups were optimized by least squares.

### Crystal data

**Table:**

C_16_H_15_N_3_O_6_	*Z* = 2
*M**_r_* = 345.31	*F*(000) = 360
Triclinic, *P*1	*D*_x_ = 1.480 Mg m^−^^3^
Hall symbol: -P 1	Mo *K*α radiation, λ = 0.71073 Å
*a* = 8.2587 (3) Å	Cell parameters from 2633 reflections
*b* = 8.7679 (4) Å	θ = 1.9–26.4°
*c* = 10.9346 (5) Å	µ = 0.12 mm^−^^1^
α = 101.616 (2)°	*T* = 296 K
β = 90.426 (2)°	Block, colourless
γ = 91.930 (2)°	0.30 × 0.25 × 0.20 mm
*V* = 775.05 (6) Å^3^	

### Data collection

**Table:**

Bruker SMART APEXII CCD diffractometer	3161 independent reflections
Radiation source: fine-focus sealed tube	2633 reflections with *I* > 2σ(*I*)
Graphite monochromator	*R*_int_ = 0.026
ω and φ scans	θ_max_ = 26.4°, θ_min_ = 1.9°
Absorption correction: multi-scan (*SADABS*; Bruker, 2008)	*h* = −10→10
*T*_min_ = 0.966, *T*_max_ = 0.977	*k* = −10→10
10840 measured reflections	*l* = −11→13

### Refinement

**Table:**

Refinement on *F*^2^	Primary atom site location: structure-invariant direct methods
Least-squares matrix: full	Secondary atom site location: difference Fourier map
*R*[*F*^2^ > 2σ(*F*^2^)] = 0.044	Hydrogen site location: inferred from neighbouring sites
*wR*(*F*^2^) = 0.126	H atoms treated by a mixture of independent and constrained refinement
*S* = 1.04	*w* = 1/[σ^2^(*F*_o_^2^) + (0.0616*P*)^2^ + 0.2861*P*] where *P* = (*F*_o_^2^ + 2*F*_c_^2^)/3
3161 reflections	(Δ/σ)_max_ = 0.001
230 parameters	Δρ_max_ = 0.24 e Å^−^^3^
1 restraint	Δρ_min_ = −0.31 e Å^−^^3^

### Special details

**Table:**

Geometry. All e.s.d.'s (except the e.s.d. in the dihedral angle between two l.s. planes) are estimated using the full covariance matrix. The cell e.s.d.'s are taken into account individually in the estimation of e.s.d.'s in distances, angles and torsion angles; correlations between e.s.d.'s in cell parameters are only used when they are defined by crystal symmetry. An approximate (isotropic) treatment of cell e.s.d.'s is used for estimating e.s.d.'s involving l.s. planes.
Refinement. Refinement of *F*^2^ against ALL reflections. The weighted *R*-factor *wR* and goodness of fit *S* are based on *F*^2^, conventional *R*-factors *R* are based on *F*, with *F* set to zero for negative *F*^2^. The threshold expression of *F*^2^ > σ(*F*^2^) is used only for calculating *R*-factors(gt) *etc*. and is not relevant to the choice of reflections for refinement. *R*-factors based on *F*^2^ are statistically about twice as large as those based on *F*, and *R*- factors based on ALL data will be even larger.

### Fractional atomic coordinates and isotropic or equivalent isotropic displacement parameters (Å^2^)

**Table:**

	*x*	*y*	*z*	*U*_iso_*/*U*_eq_	
C1	0.2531 (2)	1.32368 (19)	0.26884 (15)	0.0403 (4)	
H1	0.1710	1.3834	0.3094	0.048*	
C2	0.3654 (2)	1.3910 (2)	0.20151 (16)	0.0453 (4)	
H2	0.3626	1.4961	0.1983	0.054*	
C3	0.48192 (19)	1.2977 (2)	0.13907 (15)	0.0427 (4)	
C4	0.49206 (19)	1.1433 (2)	0.14415 (16)	0.0431 (4)	
H4	0.5719	1.0831	0.1008	0.052*	
C5	0.38164 (18)	1.07952 (19)	0.21467 (15)	0.0373 (4)	
H5	0.3883	0.9756	0.2206	0.045*	
C6	0.26034 (17)	1.16853 (17)	0.27713 (13)	0.0317 (3)	
C7	0.13772 (17)	1.09405 (17)	0.35343 (14)	0.0326 (3)	
H7	0.0602	1.1724	0.3894	0.039*	
C8	0.04677 (17)	0.96034 (18)	0.27041 (13)	0.0330 (3)	
C9	−0.07332 (18)	0.9969 (2)	0.18054 (15)	0.0386 (4)	
C10	−0.1554 (2)	0.8623 (2)	0.09238 (16)	0.0456 (4)	
H10A	−0.1777	0.8934	0.0138	0.055*	
H10B	−0.2583	0.8373	0.1271	0.055*	
C11	−0.0557 (2)	0.7178 (2)	0.06734 (15)	0.0446 (4)	
H11A	0.0400	0.7369	0.0210	0.054*	
H11B	−0.1188	0.6322	0.0170	0.054*	
C12	−0.0052 (2)	0.6741 (2)	0.18949 (15)	0.0434 (4)	
H12A	−0.0992	0.6358	0.2284	0.052*	
H12B	0.0720	0.5918	0.1732	0.052*	
C13	0.06922 (18)	0.81242 (18)	0.27499 (14)	0.0349 (3)	
C14	0.23414 (18)	0.87924 (18)	0.45824 (14)	0.0337 (3)	
C15	0.21916 (18)	1.03614 (17)	0.45763 (13)	0.0336 (3)	
C16	0.3124 (3)	0.6535 (2)	0.5443 (2)	0.0685 (6)	
H16A	0.2041	0.6094	0.5407	0.103*	
H16B	0.3655	0.6397	0.6195	0.103*	
H16C	0.3714	0.6023	0.4733	0.103*	
N1	0.30687 (19)	0.81832 (17)	0.54344 (13)	0.0445 (4)	
N2	0.28580 (17)	1.14864 (16)	0.55398 (12)	0.0405 (3)	
N3	0.5987 (2)	1.3655 (3)	0.06260 (18)	0.0627 (5)	
O1	0.16935 (14)	0.76920 (13)	0.36371 (10)	0.0414 (3)	
O2	−0.10506 (16)	1.13167 (16)	0.18180 (13)	0.0544 (4)	
O3	0.36526 (17)	1.11096 (15)	0.64145 (11)	0.0535 (3)	
O4	0.26560 (19)	1.28774 (14)	0.55197 (12)	0.0579 (4)	
O5	0.6313 (2)	1.5047 (2)	0.09323 (18)	0.0934 (6)	
O6	0.6534 (2)	1.2794 (2)	−0.02896 (18)	0.0874 (6)	
H1A	0.349 (4)	0.891 (3)	0.607 (2)	0.105*	

### Atomic displacement parameters (Å^2^)

**Table:**

	*U*^11^	*U*^22^	*U*^33^	*U*^12^	*U*^13^	*U*^23^
C1	0.0438 (9)	0.0379 (8)	0.0401 (8)	0.0039 (7)	0.0024 (7)	0.0098 (7)
C2	0.0522 (10)	0.0400 (9)	0.0465 (9)	−0.0063 (7)	−0.0046 (8)	0.0169 (7)
C3	0.0304 (8)	0.0616 (11)	0.0399 (9)	−0.0091 (7)	−0.0057 (6)	0.0211 (8)
C4	0.0287 (7)	0.0606 (11)	0.0427 (9)	0.0075 (7)	0.0012 (6)	0.0160 (8)
C5	0.0337 (8)	0.0403 (8)	0.0403 (8)	0.0053 (6)	−0.0028 (6)	0.0130 (7)
C6	0.0303 (7)	0.0371 (8)	0.0286 (7)	−0.0006 (6)	−0.0050 (5)	0.0092 (6)
C7	0.0314 (7)	0.0348 (8)	0.0330 (7)	0.0036 (6)	0.0009 (6)	0.0098 (6)
C8	0.0266 (7)	0.0424 (8)	0.0315 (7)	−0.0011 (6)	−0.0005 (6)	0.0118 (6)
C9	0.0273 (7)	0.0544 (10)	0.0384 (8)	0.0018 (6)	0.0008 (6)	0.0195 (7)
C10	0.0316 (8)	0.0687 (12)	0.0383 (9)	−0.0011 (7)	−0.0075 (7)	0.0161 (8)
C11	0.0370 (8)	0.0606 (11)	0.0343 (8)	−0.0054 (7)	−0.0050 (6)	0.0064 (7)
C12	0.0449 (9)	0.0439 (9)	0.0401 (9)	−0.0054 (7)	−0.0076 (7)	0.0071 (7)
C13	0.0308 (7)	0.0435 (9)	0.0315 (7)	−0.0017 (6)	−0.0045 (6)	0.0109 (6)
C14	0.0327 (7)	0.0401 (8)	0.0289 (7)	0.0003 (6)	−0.0026 (6)	0.0086 (6)
C15	0.0346 (7)	0.0383 (8)	0.0281 (7)	−0.0011 (6)	−0.0003 (6)	0.0077 (6)
C16	0.0977 (17)	0.0461 (11)	0.0639 (13)	0.0088 (11)	−0.0315 (12)	0.0169 (9)
N1	0.0549 (9)	0.0423 (8)	0.0374 (7)	0.0031 (6)	−0.0136 (6)	0.0110 (6)
N2	0.0481 (8)	0.0424 (8)	0.0307 (7)	−0.0034 (6)	−0.0006 (6)	0.0073 (5)
N3	0.0397 (8)	0.0917 (14)	0.0665 (11)	−0.0157 (9)	−0.0052 (8)	0.0423 (10)
O1	0.0487 (7)	0.0369 (6)	0.0386 (6)	0.0019 (5)	−0.0142 (5)	0.0083 (5)
O2	0.0471 (7)	0.0585 (8)	0.0637 (8)	0.0068 (6)	−0.0117 (6)	0.0261 (6)
O3	0.0675 (8)	0.0560 (8)	0.0356 (6)	−0.0060 (6)	−0.0162 (6)	0.0079 (5)
O4	0.0883 (10)	0.0372 (7)	0.0461 (7)	−0.0028 (6)	−0.0066 (7)	0.0044 (5)
O5	0.0823 (12)	0.1065 (14)	0.0976 (13)	−0.0536 (11)	−0.0110 (10)	0.0455 (11)
O6	0.0651 (10)	0.1239 (16)	0.0858 (12)	0.0141 (10)	0.0369 (9)	0.0481 (11)

### Geometric parameters (Å, º)

**Table:**

C1—C2	1.378 (2)	C10—H10B	0.9700
C1—C6	1.385 (2)	C11—C12	1.521 (2)
C1—H1	0.9300	C11—H11A	0.9700
C2—C3	1.379 (3)	C11—H11B	0.9700
C2—H2	0.9300	C12—C13	1.486 (2)
C3—C4	1.371 (3)	C12—H12A	0.9700
C3—N3	1.469 (2)	C12—H12B	0.9700
C4—C5	1.374 (2)	C13—O1	1.3877 (18)
C4—H4	0.9300	C14—N1	1.314 (2)
C5—C6	1.388 (2)	C14—O1	1.3569 (18)
C5—H5	0.9300	C14—C15	1.387 (2)
C6—C7	1.528 (2)	C15—N2	1.386 (2)
C7—C15	1.501 (2)	C16—N1	1.449 (2)
C7—C8	1.505 (2)	C16—H16A	0.9600
C7—H7	0.9800	C16—H16B	0.9600
C8—C13	1.327 (2)	C16—H16C	0.9600
C8—C9	1.479 (2)	N1—H1A	0.906 (10)
C9—O2	1.216 (2)	N2—O4	1.2411 (18)
C9—C10	1.504 (2)	N2—O3	1.2598 (18)
C10—C11	1.514 (3)	N3—O5	1.219 (3)
C10—H10A	0.9700	N3—O6	1.226 (3)
			
C2—C1—C6	121.08 (15)	C10—C11—H11A	109.6
C2—C1—H1	119.5	C12—C11—H11A	109.6
C6—C1—H1	119.5	C10—C11—H11B	109.6
C3—C2—C1	117.90 (16)	C12—C11—H11B	109.6
C3—C2—H2	121.1	H11A—C11—H11B	108.1
C1—C2—H2	121.1	C13—C12—C11	109.88 (14)
C4—C3—C2	122.65 (15)	C13—C12—H12A	109.7
C4—C3—N3	118.58 (17)	C11—C12—H12A	109.7
C2—C3—N3	118.77 (17)	C13—C12—H12B	109.7
C3—C4—C5	118.53 (15)	C11—C12—H12B	109.7
C3—C4—H4	120.7	H12A—C12—H12B	108.2
C5—C4—H4	120.7	C8—C13—O1	122.42 (13)
C4—C5—C6	120.72 (15)	C8—C13—C12	126.12 (14)
C4—C5—H5	119.6	O1—C13—C12	111.46 (13)
C6—C5—H5	119.6	N1—C14—O1	112.44 (13)
C1—C6—C5	119.08 (14)	N1—C14—C15	127.25 (14)
C1—C6—C7	121.28 (13)	O1—C14—C15	120.30 (13)
C5—C6—C7	119.64 (13)	N2—C15—C14	120.33 (13)
C15—C7—C8	109.22 (12)	N2—C15—C7	116.49 (13)
C15—C7—C6	111.43 (12)	C14—C15—C7	123.14 (13)
C8—C7—C6	110.03 (12)	N1—C16—H16A	109.5
C15—C7—H7	108.7	N1—C16—H16B	109.5
C8—C7—H7	108.7	H16A—C16—H16B	109.5
C6—C7—H7	108.7	N1—C16—H16C	109.5
C13—C8—C9	119.05 (14)	H16A—C16—H16C	109.5
C13—C8—C7	122.96 (13)	H16B—C16—H16C	109.5
C9—C8—C7	117.99 (13)	C14—N1—C16	125.65 (15)
O2—C9—C8	120.00 (15)	C14—N1—H1A	112.9 (19)
O2—C9—C10	122.51 (14)	C16—N1—H1A	121.4 (19)
C8—C9—C10	117.46 (14)	O4—N2—O3	120.65 (13)
C9—C10—C11	113.25 (13)	O4—N2—C15	118.39 (14)
C9—C10—H10A	108.9	O3—N2—C15	120.96 (14)
C11—C10—H10A	108.9	O5—N3—O6	124.64 (19)
C9—C10—H10B	108.9	O5—N3—C3	117.5 (2)
C11—C10—H10B	108.9	O6—N3—C3	117.88 (19)
H10A—C10—H10B	107.7	C14—O1—C13	119.94 (12)
C10—C11—C12	110.42 (14)		
			
C6—C1—C2—C3	2.2 (2)	C7—C8—C13—O1	5.2 (2)
C1—C2—C3—C4	−1.6 (3)	C9—C8—C13—C12	5.8 (2)
C1—C2—C3—N3	177.53 (15)	C7—C8—C13—C12	−174.55 (15)
C2—C3—C4—C5	−0.2 (2)	C11—C12—C13—C8	22.5 (2)
N3—C3—C4—C5	−179.32 (15)	C11—C12—C13—O1	−157.33 (14)
C3—C4—C5—C6	1.4 (2)	N1—C14—C15—N2	0.4 (2)
C2—C1—C6—C5	−1.0 (2)	O1—C14—C15—N2	−179.78 (13)
C2—C1—C6—C7	178.99 (14)	N1—C14—C15—C7	178.06 (15)
C4—C5—C6—C1	−0.8 (2)	O1—C14—C15—C7	−2.1 (2)
C4—C5—C6—C7	179.15 (14)	C8—C7—C15—N2	−169.66 (12)
C1—C6—C7—C15	−118.64 (15)	C6—C7—C15—N2	68.58 (17)
C5—C6—C7—C15	61.39 (18)	C8—C7—C15—C14	12.6 (2)
C1—C6—C7—C8	120.07 (15)	C6—C7—C15—C14	−109.17 (16)
C5—C6—C7—C8	−59.91 (17)	O1—C14—N1—C16	−3.4 (3)
C15—C7—C8—C13	−14.1 (2)	C15—C14—N1—C16	176.41 (19)
C6—C7—C8—C13	108.47 (16)	C14—C15—N2—O4	−177.86 (14)
C15—C7—C8—C9	165.53 (12)	C7—C15—N2—O4	4.3 (2)
C6—C7—C8—C9	−71.87 (16)	C14—C15—N2—O3	2.3 (2)
C13—C8—C9—O2	174.08 (15)	C7—C15—N2—O3	−175.54 (14)
C7—C8—C9—O2	−5.6 (2)	C4—C3—N3—O5	−152.56 (18)
C13—C8—C9—C10	−4.3 (2)	C2—C3—N3—O5	28.3 (2)
C7—C8—C9—C10	176.08 (13)	C4—C3—N3—O6	29.2 (2)
O2—C9—C10—C11	155.85 (16)	C2—C3—N3—O6	−150.02 (19)
C8—C9—C10—C11	−25.9 (2)	N1—C14—O1—C13	171.08 (13)
C9—C10—C11—C12	53.46 (19)	C15—C14—O1—C13	−8.8 (2)
C10—C11—C12—C13	−50.56 (19)	C8—C13—O1—C14	7.4 (2)
C9—C8—C13—O1	−174.42 (13)	C12—C13—O1—C14	−172.82 (14)

### Hydrogen-bond geometry (Å, º)

Cg1 is the centroid of the C1–C6 ring.

**Table:**

*D*—H···*A*	*D*—H	H···*A*	*D*···*A*	*D*—H···*A*
N1—H1*A*···O3	0.90 (3)	1.89 (3)	2.601 (2)	134 (2)
C11—H11*A*···O6^i^	0.97	2.54	3.352 (2)	141
C11—H11*B*···O5^ii^	0.97	2.54	3.186 (2)	124
C10—H10*A*···*Cg*1^iii^	0.97	2.90	3.515 (2)	135
C16—H16*B*···*Cg*1^iv^	0.96	2.90	3.577 (3)	142

Symmetry codes: (i) −*x*+1, −*y*+2, −*z*; (ii) *x*−1, *y*−1, *z*; (iii) −*x*, −*y*+2, −*z*; (iv) −*x*+1, −*y*+2, −*z*+1.
